# ANOVA simultaneous component analysis: A tutorial review

**DOI:** 10.1016/j.acax.2020.100061

**Published:** 2020-10-06

**Authors:** Carlo Bertinetto, Jasper Engel, Jeroen Jansen

**Affiliations:** a Department of Analytical Chemistry, Institute of Molecular Materials, Radboud University, the Netherlands; b Biometris, Wageningen UR, Droevendaalsesteeg 1, 6708 PB, Wageningen, the Netherlands

**Keywords:** Multivariate models, Design of experiments, Analysis of variance, Significance testing, Main effects, Interactions

## Abstract

When analyzing experimental chemical data, it is often necessary to incorporate the structure of the study design into the chemometric/statistical models to effectively address the research questions of interest. ANOVA-Simultaneous Component Analysis (ASCA) is one of the most prominent methods to include such information in the quantitative analysis of multivariate data, especially when the number of variables is large. This tutorial review intends to explain in a simple way how ASCA works, how it is operated and how to correctly interpret ASCA results, with approachable mathematical and visual descriptions. Two examples are given: the first, a simulated chemical reaction, serves to illustrate the ASCA steps and the second, from a real chemical ecology data set, the interpretation of results. An overview of methods closely related to ASCA is also provided, pointing out their differences and scope, to give a wide-ranging picture of the available options to build multivariate models that take experimental design into account.

## Highlights

• ASCA is a multivariate method for analysis of multi-factor data.
• An overview of the (mathematical) principles of ASCA is presented.
• Key aspects for practical application of ASCA are discussed.
• Detailed explanation of ASCA output in terms of score and loading plots is given.
• Literature review of other multivariate techniques for analysis of multi-factor data.

## Graphical abstract

**Figure:**
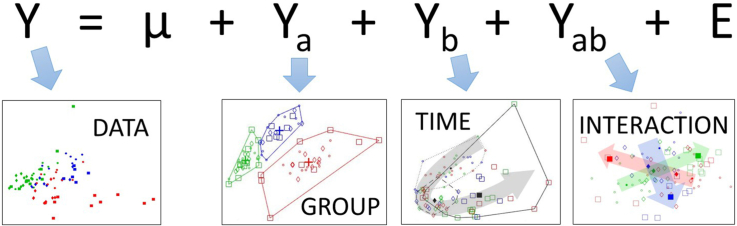

## 1 Introduction

The advances in analytical techniques seen during the last few decades have produced a spectacular increase in the amount and complexity of chemical and biological measurements at our disposal [1]. Consequently, the need for methods to correctly interpret and extract information from this wealth of data has become an ever more pressing problem for which recent advances in chemometrics, statistical learning, machine learning or artificial intelligence have proven very useful. The most typical questions addressed by a scientific study concern the relationship between the measured signals and certain groups of observations considered in the study design. In this context, more information can be extracted by including knowledge about the variation-generating mechanisms in the data, *i.e.* experimental factors and interactions related to the experimental questions and to random aspects beyond experimental control [2].

Design of Experiments (DoE) is an essential component of almost all fields of science including analytical chemistry and chemometrics [3]. It is used to identify sources of variation in the data in terms of factors that were included in the experiment and test their significance. Randomization of the different experiments mitigates the effect of possible confounding, and (causal) relationships between experimental factors and the values of measured variables can be established. For example, consider a simple theoretical case investigating a chemical reaction, particularly how three temperatures (20, 50 and 100 C) and two different catalysts (A and B) affect the yield of two final products (*y*_*1*_ and *y*_*2*_). The questions purposely addressed by this hypothetical experiment are:

(1) What is the overall effect of *temperature* on product yield?
(2) What is the effect of choosing a different *catalyst*?
(3) Is the effect of *temperature* different for each *catalyst*, i.e. is there an interaction between temperature and catalyst?

**Fig. 1:**
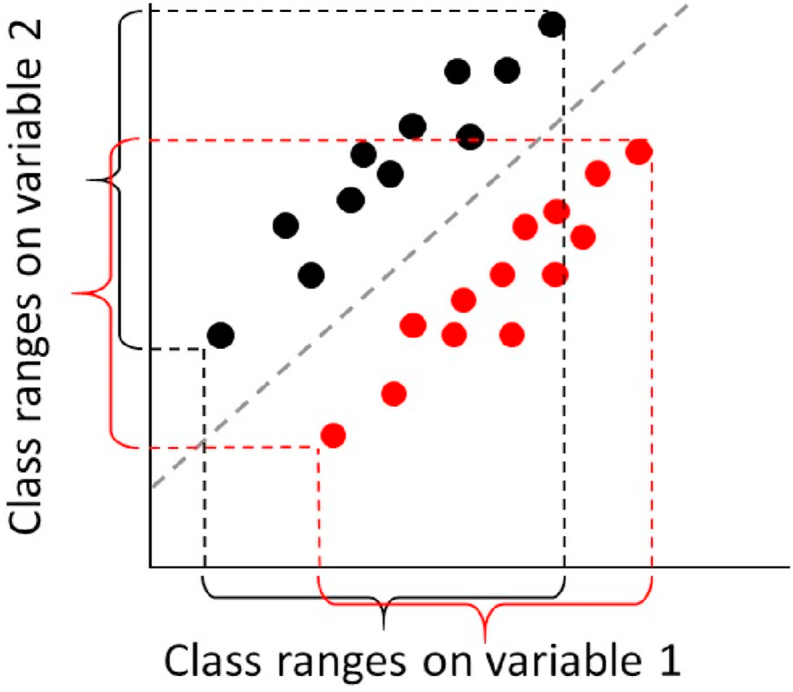
Illustration of the concept of multivariate advantage. In the plot above, no single variable can discriminate between the black and the red group, but a linear combination of both variables can separate them perfectly. (For interpretation of the references to color in this figure legend, the reader is referred to the Web version of this article.)

The most common way to address these questions is by Analysis of Variance (ANOVA), which allows for exploring the relationships between controlled factors in an experiment and a single response. In particular, it can formally separate the variability in response across the different samples into the different contributions to the experimental design, *i.e.* whether the yield of a product changes significantly with chosen *temperature* (main effect), used *catalyst* (main effect) or whether the *temperature* response is different between *catalysts* (interaction effect) [4]. However, dealing with one response at a time may be a suboptimal approach, as the yield of each product may not be separately significant with ANOVA, but may contribute to a characteristic pattern when all responses are considered simultaneously. This property is referred to as the ‘Multivariate Advantage’ [5] and is illustrated in Fig. 1.

Standard multivariate methods in chemometrics such as Principal Component Analysis (PCA) and Partial Least Squares (PLS) generally do not find the multivariate patterns answering questions (1–3) in a direct and quantitative way (note that we are not referring to the use of PLS to calculate the size of the effects in a DoE, as typically done when studying univariate responses [7]). PCA finds linear combinations of response variables that encompass the highest amount of variance observed between all samples, regardless of which temperature/catalyst group they belong to; such group information needs to be obtained from *post hoc* interpretation of the model results. Although this dimension reduction capability can be extremely useful, especially when dealing with high-dimensional and strongly collinear data (e.g. –omics studies), the individual Principal Components (PCs) do not explicitly contain resolved information on the factors *temperature* and *catalyst* (questions 1–2) and their interaction (question 3). Notably, the first few PCs may not capture any effect of the experimental factors at all. Consequently, in this context PCA tends to be used only for an initial exploration of the data.

It is possible to include aspects of the experimental design by conducting a Discriminant Analysis, by using the analytical data as the set of predictors and the class labels (of a single factor) as response. There are several Discriminant Analysis methods available, of which PLS-DA is undoubtedly the most widely used in chemometrics, able to resolve variability between groups that may not be revealed by unsupervised PCA analyses [6]. In most cases, PLS-DA is employed for binary classification of case-control studies, although extension to multi-class problems is possible. This approach allows the study of differences in product yields between *temperatures* (question 1) or *catalysts* (question 2) or between all combinations of *temperature* and *catalyst*. However, the simple observation of differences between all groups does not enable a more holistic result that also includes the differences in *temperature*-dependence of the response between the different *catalysts* (question 3), unless very specific contrasts are subsequently studied. Such objectives require the formal introduction of relationships between the different groups of observations.

If the studied factors are of a crossed nature, i.e. every level of one factor occurs at least once for every level of another factor, these relationships can be established by combining aspects of ANOVA and multivariate data analysis. A multivariate extension of ANOVA, called Multivariate ANOVA (MANOVA) [8], has been around for nearly a century [9], see Section 4.1. Analogous to PLS, this method also involves a dimension reduction step to highlight differences between the experimental groups (now specifically focused on main and interaction effects) and identify the associated response patterns. However, MANOVA is not applicable to data with more measured variables than observations. This limitation, particularly cumbersome in e.g. -omics studies, has led to the development of other ways to interface ANOVA with multivariate dimension reduction methods.

One of such ways is by hyphenating ANOVA and PCA, so that the resulting method is applicable to multi-factor, high-dimensional data. This combination has led to a family of methods, of which the most widely known is ANOVA-Simultaneous Component Analysis (ASCA). ASCA is the focus of this review tutorial. We will detail its principles by means of analysis of simulated (Section 2) and experimental data from a chemical ecology study (Section 3). Finally, Section 4 provides an overview of closely related data analysis methods.

## 2 ANOVA simultaneous component analysis: main principles

### 2.1 The data are decomposed according to the experimental design

A detailed description of the ASCA method can be found in previous works [2,10]; here we will illustrate its application by means of a numerical example. In the hypothetical reaction study introduced above, let us indicate the number of response variables (the number of reaction products for which the yield is measured) as P, the levels of the factors *temperature* and *catalyst* as *I* (i=1,…,I) and *J* (j=1,…,J). Let N indicate the total number of observations, which for a balanced (full factorial) design, i.e. with the same number of independent observations K for each combination of levels (or *cell*), is equal to N=KxIJ. In the present case, P=2 (number of reaction products), I=3 (considered temperatures), J=2 (catalysts), K=2 (biological replicates) and N=12. Note that this is quite a limited number of observations, but here it allows us to (numerically) show all details of an ASCA analysis. Let X (size Nx P) be the matrix of all measured responses, and $x_{ijkp}$ denote the concentration of a single product p for replicate $k_{i,j}$ from temperature *j* with catalyst *i*. The simulated data used in this example are shown in Table 1.

**Table 1:** The simulated reaction data set used to explain ASCA. The data consists of the yields (*x*_*1*_ and *x*_*2*_) for two products as a function of the applied temperature and catalyst.

Exp.	Temp. (C)	Catalyst	Yield (g/l)	
			[*x*_*1*_]	[*x*_*2*_]
1	20	A	0.31	2.60
2	20	A	0.98	2.66
3	20	B	1.71	2.35
4	20	B	1.14	2.00
5	50	A	2.07	2.90
6	50	A	1.73	2.20
7	50	B	2.40	1.67
8	50	B	3.16	2.54
9	100	A	2.13	0.64
10	100	A	2.27	0.89
11	100	B	3.25	0.85
12	100	B	2.90	0.46

According to the standard ANOVA calculations, each response can be partitioned into additive effects [11,12] (for simplicity, the subscript p has been omitted from all the terms on the right-hand side):

(1) $$x_{ijkp}=\mu +\alpha_{i}+\beta_{j}+{\left(\alpha \beta \right)}_{ij}+\varepsilon_{k_{ij}},$$

where μ indicates the offset, $\alpha_{i}$ the effect of temperature, $\beta_{j}$ the effect of catalyst, ${\left(\alpha \beta \right)}_{ij}$ the interaction between temperature and catalyst, and $\varepsilon_{k_{ij}}$ the residuals. In ASCA, “sum-to-zero” constraints are imposed to ensure uniqueness of the estimated parameters, therefore all effects are described as deviations from the overall mean μ. For a balanced design, these effects can be estimated by the ANOVA formulas reported in Table A1 of the Appendix. However, it is more convenient to re-express Equation (1) into matrix notation, especially for designs involving more main effects and interactions than considered here:

(2) $$x_{p}=D\theta_{p}+\varepsilon_{p},$$

where $x_{p}$ is the Nx1 vector of observations for a single response p , matrix D of size N×q (with q the number of parameters in the linear model, see below) specifies the Design of Experiments in a ‘dummy notation’, vector $\theta_{p}$ (qx1) is the relevant set of regression coefficients for response *p* and $\varepsilon_{p}$ (size Nx1) contains the residuals [[12], [13], [14]]. In our example, with 3 temperatures and 2 catalysts evaluated for 2 replicates, matrix D is given by:

(3) $$D=\left[\begin{array}{cccccc} 1 & 1 & 0 & 1 & 1 & 0\\ 1 & 1 & 0 & 1 & 1 & 0\\ 1 & 1 & 0 & -1 & -1 & 0\\ 1 & 1 & 0 & -1 & -1 & 0\\ 1 & 0 & 1 & 1 & 0 & 1\\ 1 & 0 & 1 & 1 & 0 & 1\\ 1 & 0 & 1 & -1 & 0 & -1\\ 1 & 0 & 1 & -1 & 0 & -1\\ 1 & -1 & -1 & 1 & -1 & -1\\ 1 & -1 & -1 & 1 & -1 & -1\\ 1 & -1 & -1 & -1 & 1 & 1\\ 1 & -1 & -1 & -1 & 1 & 1 \end{array}\right]$$

Each row of D codifies the levels of the main and interaction effects used to produce each observation. The type of coding used in (3), called sum coding or deviation coding [15], is not the only possible way to express the ANOVA model, but is the most appropriate in the context of ASCA for reasons of simplicity and desired (sum-to-zero) constraints [14]. The first column refers to the global mean μ, which is the same for all observations and thus coded with a constant value chosen as 1. Columns 2 and 3 together code for factor *temperature*, while column 4 codes for factor *catalyst;* columns 5 and 6 correspond to the interaction between *temperature* and *catalyst*. In general, each main effect with z levels is coded with z−1 columns, e.g. *temperature* has three levels and hence two columns. Because of the sum-to-zero constraints imposed, the first z−1 levels are coded with zeros and ones, and level z (the last level) is coded with -1’s, e.g. the *temperature* levels are coded as $\left[\begin{array}{cc} 1 & 0 \end{array}\right]$, $\left[\begin{array}{cc} 0 & 1 \end{array}\right]$ and $\left[\begin{array}{cc} -1 & -1 \end{array}\right]$, respectively. The last two columns of D, coding for the *temperature*-*catalyst* interaction, are obtained by the row-wise Kronecker product of the columns of corresponding main-effects (in other words: element-wise multiplication of the *catalyst* column with the first and second *temperature* column, respectively).

After specifying the design (or model) matrix D, an estimate of the regression coefficients ($\hat{\theta_{p}}$) is obtained by least-squares:

(4) $$\hat{\theta_{p}}={\left(D^{T}D\right)}^{-1}D^{T}x_{p},$$

where ${\left(.\right)}^{T}$ and ${\left(.\right)}^{-1}$ indicate the transpose and inverse operators, respectively. The resulting estimates after applying this formula to e.g. the first response variable are:

(5) $$\hat{\theta_{1}}=\left[\begin{array}{c} 2.00\\ -0.97\\ 0.33\\ -0.42\\ 0.03\\ -0.02 \end{array}\right]\;$$

These regression coefficients imply that e.g. the overall mean is 2.00, temperature 20 has a yield (averaged over catalyst) lower by 0.97, temperature 20 with catalyst A has a yield 0.03 higher than if the two factors were completely independent, and so on according to the rows of (3).

Subsequently, to obtain the main and interaction effects, the relevant blocks of columns in D are multiplied with their corresponding $\hat{\theta_{p}}$ as follows [14]:

(6) $$x_{fp}=Ddiag\left(C\right)\hat{\theta_{p}}$$

where *f* ∈{a,b,ab} contains the estimates of the levels of the factor of interest; vector C (size qx1) highlights which parameters in the model (i.e. which columns of D) correspond to that factor, and diag(C) indicates a diagonal matrix constructed from C. For the reaction data set, to obtain estimates of the levels of factors α (temperature), β (catalyst) and αβ (interaction) the following indicators can be used: $C_{\alpha }=\left[0,\;1,\;1,\;0,\;0\;,0\right]$, $C_{\beta }=\left[0,0,0,1,0,0\right]$, and $C_{\alpha \beta }=\left[0,0,0,0,1,1\right]$. For instance, the estimates of temperature levels for product 1 are obtained as:

(7) $$x_{\alpha 1}=\left[\begin{array}{cccccc} 1 & 1 & 0 & 1 & 1 & 0\\ 1 & 1 & 0 & 1 & 1 & 0\\ 1 & 1 & 0 & -1 & -1 & 0\\ 1 & 1 & 0 & -1 & -1 & 0\\ 1 & 0 & 1 & 1 & 0 & 1\\ 1 & 0 & 1 & 1 & 0 & 1\\ 1 & 0 & 1 & -1 & 0 & -1\\ 1 & 0 & 1 & -1 & 0 & -1\\ 1 & -1 & -1 & 1 & -1 & -1\\ 1 & -1 & -1 & 1 & -1 & -1\\ 1 & -1 & -1 & -1 & 1 & 1\\ 1 & -1 & -1 & -1 & 1 & 1 \end{array}\right]\left[\begin{array}{cc} \begin{array}{ccc} 0 & 0 & 0\\ 0 & 1 & 0\\ 0 & 0 & 1 \end{array} & \begin{array}{ccc} 0 & 0 & 0\\ 0 & 0 & 0\\ 0 & 0 & 0 \end{array}\\ \begin{array}{ccc} 0 & 0 & 0\\ 0 & 0 & 0\\ 0 & 0 & 0 \end{array} & \begin{array}{ccc} 0 & 0 & 0\\ 0 & 0 & 0\\ 0 & 0 & 0 \end{array} \end{array}\right]\left[\begin{array}{c} 2.00\\ -0.97\\ 0.33\\ -0.42\\ 0.03\\ -0.02 \end{array}\right]=\left[\begin{array}{c} \begin{array}{c} \begin{array}{c} -0.97\\ -0.97 \end{array}\\ \begin{array}{c} -0.97\\ -0.97 \end{array} \end{array}\\ \begin{array}{c} \begin{array}{c} 0.33\\ 0.33 \end{array}\\ \begin{array}{c} 0.33\\ 0.33 \end{array} \end{array}\\ \begin{array}{c} \begin{array}{c} 0.64\\ 0.64 \end{array}\\ \begin{array}{c} 0.64\\ 0.64 \end{array} \end{array} \end{array}\right]$$

If the experimental design involves more factors (and interactions) and levels, the linear model must be modified accordingly but the subsequent steps are essentially the same. Note that the expressions given above apply to analysis of balanced designs. The treatment of unbalanced designs is further discussed in Section 2.3.

If this whole procedure is applied to all response variables (yields) and all the column vectors containing the estimates for each main effect and interaction are collected in matrices, the data matrix X is partitioned as follows [2,10,14]:

(8) $$X=X_{m}+X_{a}+X_{b}+X_{ab}+X_{e},$$

where the rows in matrix $X_{m}$ contain the sample estimates of the overall mean for each response (i.e. $X_{m}=1m^{T}$, where 1 is a column vector of ones and m is the vector of sample means); $X_{a}$ and $X_{b}$ are effect matrices with the sample estimates of the level means for factors *temperature* and *catalyst*, respectively; $X_{ab}$ contains estimates of the interaction effect between *temperature* and *catalyst* (i.e. the means of each *temperature-catalyst* combination after subtracting the means of both main effects); and matrix $X_{e}=X-D\hat{\theta }$ (with $\hat{\theta }$ indicating the effect estimates for all responses) contains the observed within-level variability. For our hypothetical reaction, this expression corresponds to:

(9) $$\begin{array}{c} X\\ \left[\begin{array}{cc} 0.31 & 2.60\\ 0.98 & 2.66\\ 1.71 & 2.35\\ 1.14 & 2.00\\ 2.07 & 2.90\\ 1.73 & 2.20\\ 2.40 & 1.67\\ 3.16 & 2.54\\ 2.13 & 0.64\\ 2.27 & 0.89\\ 3.25 & 0.85\\ 2.90 & 0.46 \end{array}\right] \end{array}=\begin{array}{c} X_{m}\\ \left[\begin{array}{cc} 2.00 & 1.81\\ 2.00 & 1.81\\ 2.00 & 1.81\\ 2.00 & 1.81\\ 2.00 & 1.81\\ 2.00 & 1.81\\ 2.00 & 1.81\\ 2.00 & 1.81\\ 2.00 & 1.81\\ 2.00 & 1.81\\ 2.00 & 1.81\\ 2.00 & 1.81 \end{array}\right] \end{array}+\begin{array}{c} X_{a}\\ \left[\begin{array}{cc} -0.97 & 0.59\\ -0.97 & 0.59\\ -0.97 & 0.59\\ -0.97 & 0.59\\ 0.33 & 0.51\\ 0.33 & 0.51\\ 0.33 & 0.51\\ 0.33 & 0.51\\ 0.63 & -1.10\\ 0.63 & -1.10\\ 0.63 & -1.10\\ 0.63 & -1.10 \end{array}\right] \end{array}+\begin{array}{c} X_{b}\\ \left[\begin{array}{cc} -0.42 & 0.17\\ -0.42 & 0.17\\ 0.42 & -0.17\\ 0.42 & -0.17\\ -0.42 & 0.17\\ -0.42 & 0.17\\ 0.42 & -0.17\\ 0.42 & -0.17\\ -0.42 & 0.17\\ -0.42 & 0.17\\ 0.42 & -0.17\\ 0.42 & -0.17 \end{array}\right] \end{array}+\begin{array}{c} X_{ab}\\ \left[\begin{array}{cc} 0.03 & 0.06\\ 0.03 & 0.06\\ -0.03 & -0.06\\ -0.03 & -0.06\\ -0.02 & 0.05\\ -0.02 & 0.05\\ 0.02 & -0.05\\ 0.02 & -0.05\\ -0.01 & -0.11\\ -0.01 & -0.11\\ 0.01 & 0.11\\ 0.01 & 0.11 \end{array}\right] \end{array}+\begin{array}{c} X_{e}\\ \left[\begin{array}{cc} -0.33 & -0.03\\ 0.33 & 0.03\\ 0.28 & 0.17\\ -0.28 & -0.17\\ 0.17 & 0.34\\ -0.17 & -0.34\\ -0.38 & -0.43\\ 0.38 & 0.43\\ -0.07 & -0.13\\ 0.07 & 0.13\\ 0.18 & 0.19\\ -0.18 & -0.19 \end{array}\right] \end{array}$$

The decomposition in (8) also partitions the sum-of-squares of the elements in X into factor-specific sums-of-squares [10]:

(10) $${||\text{X}||}^{2}={||{\text{X}}_{\text{m}}||}^{2}+{||{\text{X}}_{\text{a}}||}^{2}+{||{\text{X}}_{\text{b}}||}^{2}+{||{\text{X}}_{\text{ab}}||}^{2}+{||{\text{X}}_{\text{e}}||}^{2},$$

where ||.||^2^ indicates the squared Frobenius norm, i.e. the sum of the squared matrix elements. For the reaction data set these are equal to:

(11) 104.84=87.57+13.09+2.50+0.09+1.60,

If the design is balanced, these sums-of-squares can then be used to quantify the percentage of total variation in X that is explained by each factor and interaction [14]:

(12) $$\%\;Var\left(f\right)=\frac{X_{f}^{2}}{X^{2}-X_{m}^{2}}*100,\;f\in \left\{a,b,ab,e\right\},$$

resulting in 75.78%, 14.45%, 0.50% and 9.27% for temperature, catalyst, interaction and residuals, respectively. If, on the other hand, the design is unbalanced, the sums of squares are not uniquely defined, rendering their interpretation less straightforward (see Section 2.3).

### 2.2 PCA is applied to the decomposed data

After performing a single-response ANOVA decomposition for all variables, the second major step of ASCA consists in examining estimated effects for all variables simultaneously by applying PCA to each sub-matrix in (8) related to a factor or interaction [2,10,16]:

(13) $$X=X_{\text{m}}+T_{a}P_{a}^{T}+T_{b}P_{b}^{T}+T_{ab}P_{ab}^{T}+X_{e},$$

where T and P denote, for the corresponding factor or interaction, the scores and loadings matrices, respectively, whereas the residual term $X_{e}$ expresses the deviations of each individual replicate from the average effects accounted for in the model. To be specific, the performed operation is a Simultaneous Component Analysis [17] (SCA, hence the name ASCA), which is a generalization of PCA for the case of several populations sharing a common set of measured variables. Equation (13) is essentially another variance decomposition step, in which the largest amount of variance among the level means for a certain factor is explained by the first PC, and in decreasing order by the following ones, (just as in standard PCA). However, none of these PCA sub-models describes the whole variation between all observations, which is what would be obtained by a regular PCA applied to the original, undecomposed, data matrix. Instead, they provide information specific for every main effect or interaction, which may include very subtle effects that are normally masked by other sources of chemical or biological variability. Of course, such a deeper exploration of course requires comprehensive quantitative validation to determine the significance of the observed effects, typically, by means of a permutation-testing or bootstrap procedure [[18], [19], [20]] (see Section 2.4).

It is worth noting that each PCA submodel can have only a maximum number of PCs, corresponding to the number of parameters in the linear model (and therefore the number of columns in D) relevant to a given factor or interaction. For main effects, this corresponds to the number of factor levels – 1. For example, if the factor temperature has three levels, two PCs already explain 100% of the variance between the level means for this factor.

**Fig. 2:**
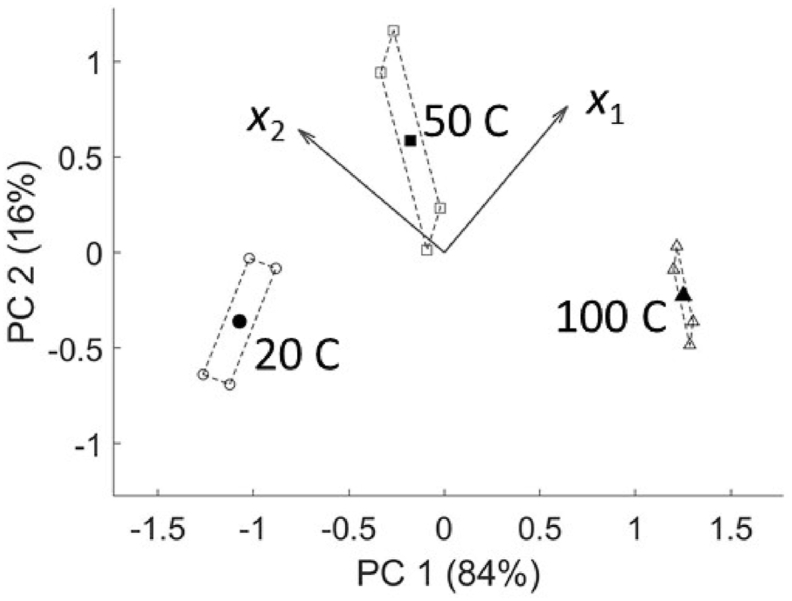
Biplot of the factor temperature for the theoretical reaction example. The filled shapes are the scores of the level averages; the empty ones (whose spread is delimited by dashed lines) are the projections of residuals from the ASCA model. The arrows are the loadings of the product yields. The numbers in parenthesis on the axis labels express the percentage of explained variance for each PC (out of the variance explained by that specific factor).

Just as in PCA, the differences between the level means of a main effect or interaction as calculated by ASCA may be visualized in a score plot [2,10]. For example, Fig. 2 visualizes the level means of the factor *temperature* by plotting the first column of $T_{a}$ (PC1) against the second (PC2). Since some difference between the group (level) means will always be observed, even if the effect is not significant, it is more informative to also project on the same plot the residuals from the data matrix decomposition ($X_{e}$) around their relevant scores [21]. For the temperature effect, that would be realized by summing up the effect and residual matrices ($X^{*}=X_{a}+X_{e}$), then projecting $X^{*}$ onto the PCs of the temperature sub-model ($T_{a}^{*}=X^{*}P_{a}$), and plotting the columns of $T_{a}^{*}$ along the scores of the level means (empty dots in Fig. 2). This procedure allows for visualizing not only the between-group (level) differences for factor temperature, but also the within-group variation in the direction of the selected PCs. It also enables a qualitative evaluation of the actual relevance of the observed between-group differences, since when the latter are large compared to the variation of the observations around their mean, the factor or interaction in question is usually significant. However, this procedure does not constitute a formal hypothesis test, which will be discussed in Section 2.4.

The response variables associated to the differences observed in the score plots are identified in each PCA sub-model by a specific set of loadings [2,10]. These may be visualized separately, or together in a biplot with the scores to see the relationship between levels and response variables in a single view. For instance, the plot in Fig. 2 shows that the extreme temperatures increase the yield of only one product (*x*_1_ or *x*_2_), while at 50 C both yields are enhanced, as its relevant score is on the positive side of both loading vectors. The same considerations in the construction of biplots apply as in normal PCA [22]. The relatively narrow range of the projected residuals compared to the spread of the level-means indicates that the temperature effect is likely to be significant (and this is confirmed more rigorously by statistical tests described below).

For data with a higher number of variables and possibly quite noisy measurements, the interpretation of loadings can easily get very cumbersome. However, several tools employed in PCA for this task are suitable in ASCA as well, such as constructing bootstrapped confidence intervals for each loading coefficient [23] or performing an implicit variable selection by applying sparse (rather than normal) PCA [24], as implemented in Group-wise ASCA (GASCA) [25]. For cases in which there is a natural ordering among the variables, e.g. spectral data, there is also the option of interval-ASCA (i-ASCA) [26], which fits several ASCA models to subsets of variables (instead of the complete data) and subsequently applies some form of multiplicity correction to take into account that multiple models are being assessed rather than a single one.

Another important similarity between ASCA and PCA is that the result is heavily influenced by the type of scaling applied to the data matrix, which can be used to focus on relative differences rather than absolute ones, see Section 3.2. However, ASCA also enables other forms of scaling related to the design information. Most notably, the effect matrices in (8) can be scaled with the standard deviations of the residual effect matrix $X_{e}$, in order to highlight variables with large between-group variance as compared to the within-group one [12,27]. It is also possible to take into account the within-group correlations between the responses when reducing the dimension of the data [8,28], which may even better highlight multivariate effects attributable to a factor or interaction. Furthermore, scaling with respect to a reference group is also possible [27].

### 2.3 Additional corrections are needed for unbalanced data

Although the least squares estimators obtained in (4), (5) are generally unbiased, unbalanced data may nevertheless affect the subsequent dimension reduction by PCA. If certain cells in the experimental design are over- or underrepresented, the resulting effect matrices are not completely orthogonal to each other. Consequently, their PCs do not necessarily describe variation solely due to the considered factor and (10) is no longer valid.

It might appear that the easiest solution could be to simply rebalance the design by removing observations. However, it is often far from obvious how the design should be rebalanced, especially in complicated designs; this approach also leads to the loss of useful information and a reduction in statistical power. Alternatively, the sums of squares correction methods already used in ANOVA were extended to ASCA [12,14,29]. The simplest one, known as “type-I sums of squares” fits the model sequentially: e.g. factor *temperature*, followed by factor *catalyst*, followed by their interaction. However, for unbalanced data the resulting sums of squares attributed to a factor and the PCA output are influenced by the order in which the main effects enter the model [30]. As demonstration, let us add a single data point to the reaction data set, with temperature 20 C, catalyst A, *x*_*1*_ = 1.23 and *x*_*2*_ = 2.98. After subtracting the overall mean, the regression coefficients for the factor temperature can be computed by using in (4) only the relevant columns of the design matrix:

(14) $$\hat{\theta_{\alpha }}={\left({\left[\begin{array}{cc} d_{*,2} & d_{*,3} \end{array}\right]}^{T}\left[\begin{array}{cc} d_{*,2} & d_{*,3} \end{array}\right]\right)}^{-1}{\left[\begin{array}{cc} d_{*,2} & d_{*,3} \end{array}\right]}^{T}\left[\begin{array}{cc} -1.63 & 0.70\\ -0.96 & 0.76\\ -0.23 & 0.45\\ -0.80 & 0.10\\ 0.13 & 1.00\\ -0.21 & 0.30\\ 0.46 & -0.23\\ 1.22 & 0.64\\ 0.19 & -1.26\\ 0.33 & -1.01\\ 1.31 & -1.05\\ \begin{array}{c} 0.96\\ -0.71 \end{array} & \begin{array}{c} -1.44\\ 1.08 \end{array} \end{array}\right]=\left[\begin{array}{cc} -0.93 & 0.66\\ 0.32 & 0.48 \end{array}\right]$$

where $\left[\begin{array}{cc} d_{*,2} & d_{*,3} \end{array}\right]$ denotes the second and third column of D. The corresponding effect matrices can be calculated as in (6), filling the regression coefficients of other factors with zeros:

(15) $$X_{a}^{’}=\left[\begin{array}{cccccc} 1 & 1 & 0 & 1 & 1 & 0\\ 1 & 1 & 0 & 1 & 1 & 0\\ 1 & 1 & 0 & -1 & -1 & 0\\ 1 & 1 & 0 & -1 & -1 & 0\\ 1 & 0 & 1 & 1 & 0 & 1\\ 1 & 0 & 1 & 1 & 0 & 1\\ 1 & 0 & 1 & -1 & 0 & -1\\ 1 & 0 & 1 & -1 & 0 & -1\\ 1 & -1 & -1 & 1 & -1 & -1\\ 1 & -1 & -1 & 1 & -1 & -1\\ 1 & -1 & -1 & -1 & 1 & 1\\ \begin{array}{c} 1\\ 1 \end{array} & \begin{array}{c} -1\\ 1 \end{array} & \begin{array}{c} -1\\ 0 \end{array} & \begin{array}{c} -1\\ 1 \end{array} & \begin{array}{c} 1\\ 1 \end{array} & \begin{array}{c} 1\\ 0 \end{array} \end{array}\right]\left[\begin{array}{cc} \begin{array}{ccc} 0 & 0 & 0\\ 0 & 1 & 0\\ 0 & 0 & 1 \end{array} & \begin{array}{ccc} 0 & 0 & 0\\ 0 & 0 & 0\\ 0 & 0 & 0 \end{array}\\ \begin{array}{ccc} 0 & 0 & 0\\ 0 & 0 & 0\\ 0 & 0 & 0 \end{array} & \begin{array}{ccc} 0 & 0 & 0\\ 0 & 0 & 0\\ 0 & 0 & 0 \end{array} \end{array}\right]\left[\begin{array}{cc} \begin{array}{c} 0\\ -0.93\\ 0.32 \end{array} & \begin{array}{c} 0\\ 0.66\\ 0.48 \end{array}\\ \begin{array}{c} 0\\ 0\\ 0 \end{array} & \begin{array}{c} 0\\ 0\\ 0 \end{array} \end{array}\right]$$

The resulting effect matrices for temperature and catalyst factors, the latter calculated in analogous way from the residual of the previous calculation, are:

(16) $$X_{a}^{’}=\left[\begin{array}{cc} -0.93 & 0.66\\ -0.93 & 0.66\\ -0.93 & 0.66\\ -0.93 & 0.66\\ 0.32 & 0.48\\ 0.32 & 0.48\\ 0.32 & 0.48\\ 0.32 & 0.48\\ 0.62 & -1.14\\ 0.62 & -1.14\\ 0.62 & -1.14\\ \begin{array}{c} 0.62\\ -0.93 \end{array} & \begin{array}{c} -1.14\\ 0.66 \end{array} \end{array}\right]\;X_{b}^{’}=\left[\begin{array}{cc} -0.37 & 0.19\\ -0.37 & 0.19\\ 0.37 & -0.19\\ 0.37 & -0.19\\ -0.37 & 0.19\\ -0.37 & 0.19\\ 0.37 & -0.19\\ 0.37 & -0.19\\ -0.37 & 0.19\\ -0.37 & 0.19\\ 0.37 & -0.19\\ \begin{array}{c} 0.37\\ -0.37 \end{array} & \begin{array}{c} -0.19\\ 0.19 \end{array} \end{array}\right]$$

whose sum-of-squares are 14.54 and 2.27, respectively. On the other hand, applying the same procedure first to the catalyst factor, then temperature, yields:

(17) $$X_{a}^{’’}=\left[\begin{array}{cc} -0.87 & 0.62\\ -0.87 & 0.62\\ -0.87 & 0.62\\ -0.87 & 0.62\\ 0.29 & 0.50\\ 0.29 & 0.50\\ 0.29 & 0.50\\ 0.29 & 0.50\\ 0.58 & -1.12\\ 0.58 & -1.12\\ 0.58 & -1.12\\ \begin{array}{c} 0.58\\ -0.87 \end{array} & \begin{array}{c} -1.12\\ 0.62 \end{array} \end{array}\right]\;X_{b}^{’’}=\left[\begin{array}{cc} -0.45 & 0.24\\ -0.45 & 0.24\\ 0.45 & -0.24\\ 0.45 & -0.24\\ -0.45 & 0.24\\ -0.45 & 0.24\\ 0.45 & -0.24\\ 0.45 & -0.24\\ -0.45 & 0.24\\ -0.45 & 0.24\\ 0.45 & -0.24\\ \begin{array}{c} 0.45\\ -0.45 \end{array} & \begin{array}{c} -0.24\\ 0.24 \end{array} \end{array}\right]$$

with sum-of-squares of 13.43 and 3.31. The discrepancy between these two outcomes, corresponding to about 6% of explained variance, is expected to be even larger for data sets with greater imbalance.

An alternative that avoids this ambiguity consists in calculating corrected effect matrices $X^{\prime \prime \prime }$, defined as the difference between the residual matrix of a reduced model that excluded certain columns of D from (2), and the residual matrix of a full model that contained all effects [31]. When the correction concerns only main effects, it is known as “type-II sums of squares”, whereas it is denoted “type-III” if it also corrects for interactions. For example, $X_{a}^{\prime \prime \prime }$ in the latter case describes the effect of *temperature* given the factor *catalyst* and the interaction *temperature* x *catalyst*, i.e. $X_{\left(a|b,ab\right)}$, obtained by:

(18) $$X_{a}^{\prime \prime \prime }=X_{\left(a|b,ab\right)}=X_{e}^{\left(a\right)}-X_{e}=X-{\hat{X}}^{\left(a\right)}-\left(X-\hat{X}\right)=\hat{X}-{\hat{X}}^{\left(a\right)}=\;D\hat{\theta }-D^{\left(a\right)}\hat{\theta^{\left(a\right)}}$$

where $X_{e}$ is the residuals matrix from (8), $X_{e}^{\left(a\right)}$ the residuals matrix of the reduced model without factor *a*, $\hat{X}$ and ${\hat{X}}^{\left(a\right)}$ the data matrices estimated by the full and reduced model, respectively, D is the design matrix (with thirteen rows to account for the additional data point in this unbalanced design), $D^{\left(a\right)}=Ddiag\left(\left[1,\;0,\;0,\;1,\;1,\;1\right]\right)$, i.e. a design matrix in which the second and third columns are replaced by zeros, and $\hat{\theta }$ and $\hat{\theta^{\left(a\right)}}$ the regression coefficients for the full and reduced model, obtained as in (4) using D and $D^{\left(a\right)}$, respectively. The resulting corrected effect matrices for temperature and catalyst are:

(19) $$X_{a}^{’’’}=\left[\begin{array}{cc} -0.68 & 0.47\\ -0.68 & 0.47\\ -1.02 & 0.71\\ -1.02 & 0.71\\ 0.36 & 0.46\\ 0.36 & 0.46\\ 0.36 & 0.46\\ 0.36 & 0.46\\ 0.66 & -1.16\\ 0.66 & -1.16\\ 0.66 & -1.16\\ \begin{array}{c} 0.66\\ -0.68 \end{array} & \begin{array}{c} -1.16\\ 0.47 \end{array} \end{array}\right]\;X_{b}^{’’’}=\left[\begin{array}{cc} -0.28 & 0.13\\ -0.28 & 0.13\\ 0.41 & -0.20\\ 0.41 & -0.20\\ -0.41 & 0.20\\ -0.41 & 0.20\\ 0.41 & -0.20\\ 0.41 & -0.20\\ -0.41 & 0.20\\ -0.41 & 0.20\\ 0.41 & -0.20\\ \begin{array}{c} 0.41\\ -0.28 \end{array} & \begin{array}{c} -0.20\\ 0.13 \end{array} \end{array}\right]$$

whose sums-of-squares are 13.59 and 2.38, respectively. Notice that this correction causes some rows, e.g. rows 1–4 of $X_{a}^{\prime \prime \prime }$, to have slightly different values even if they refer to the same level of the considered factor.

Thiel et al. [14] proposed an algorithm using type III sum-of-squares, known as ASCA+, and showed that it corrects the bias of the effects that appear in conventional ASCA, especially for interaction terms. In particular, regular ASCA applied to unbalanced data tends to make non-significant interactions appear as significant. Later, Martin and Govaerts further generalized this approach to linear mixed models, in a method named LiMM-PCA [32]. Among its main differences with ASCA are an initial PCA transformation to de-correlate the responses and the use of the effects covariance matrix for the random effects, whose parameters are estimated by Restricted Maximum Likelihood. This algorithm ought to be considered when the experimental design contains both fixed and random effects.

### 2.4 The statistical significance of effects can be tested in different ways

The incorporation of external knowledge on the experimental design, in the form of the design matrix D, confers ASCA a supervised nature. Consequently, before interpreting the obtained scores and loadings it is paramount to rigorously assess the significance of any factor, to ensure that the result is not produced by overfitting. Analogously to ANOVA, significant effects are defined as those for which a clear difference is observed in at least one of the levels. The most common significance testing involves resampling methods like bootstrap and permutation.

Bootstrapping works by substituting a few samples with repetitions of others in the same data set, i.e. random sampling of observations with replacement, while maintaining an identical data set size and groupings (factors and levels) in the experimental design. This method allows to determine not only the significance of the whole model, but also the confidence intervals for scores and loading parameters, which help in determining which response variables are significant. Performing this calculation requires dealing with the inherent non-uniqueness of PCA, for example by re-ordering and applying a Procrustes rotation to the bootstrapped PCs to align them to the components obtained from all data [33]. Care must also be placed on the resampling scheme, such that the structure of the experimental design remains intact [20]. For the final calculation of the confidence limits from the estimated model parameter distributions, several authors recommend the bias-corrected and accelerated method [20,34].

However, Vis et al. argue that the bootstrapping is not the most reliable method to estimate the standard deviation of the difference between level means without making extra assumptions [18]. On the other hand, permutation tests randomly permute the factor levels, usually by reshuffling the rows of D, and recalculate the level-mean differences every time. This procedure thus generates null-distributions of a certain metric for each factor or interaction, which can be compared to the corresponding values of the real model. Different metrics can be employed for this purpose, often borrowed from the MANOVA literature, such as the sums-of-squares (SSQ) of the effect matrix (as defined in (9)) [18] or Wilk’s lambda statistic [12,35]. After performing a large number of permutations, typically between 1000 and 10000, the p-value of the test is defined as the fraction of permutations for which the employed metric was better (i.e. higher or lower depending on which metric) than the unpermuted one. An effect is considered significant if its p-value is smaller than an appropriate significance threshold, e.g. the commonly employed 0.05. It is important to note that permutation tests are exact only for main effects, but approximate tests for interactions have nonetheless been developed [18,19]. Other special cases, such as nested designs, are also considered in the literature [19].

In the case of balanced experimental designs, it is also possible to calculate (within-group) confidence ellipses in score plots [36], based on multivariate distributional theory. One advantage of this method as compared to permutation tests is the possibility to make direct pairwise comparisons between different factor levels. The assumptions required to estimate these ellipsoids may not be very often fulfilled, but the authors claim that the approach is still effective in cases of slight unbalancedness, especially if used as an explorative tool.

## 3 Example: analysis of a chemical ecology data set

The second example of this paper illustrates the possibilities of ASCA in further detail, especially concerning the interpretation of results. It consists of a well-known chemical ecology data set that examines how feral *Brassica oleracea* responds to Jasmonic Acid, a model hormone treatment to simulate herbivory [37]. This hormone was applied to either the roots or to the shoots of the plant, and the dynamic response was measured 1, 7 and 14 days after the treatment. To characterize this response, thirteen compounds with ecological function known as ‘glucosinolates’ were measured at each time point. The measurement was destructive, so a different plant was analysed in each case. Because of the removal of outlying samples, the resulting experimental design is unbalanced and each cell contains 6–10 observations.

It is worth noting that the full potential of ASCA would be better displayed on a data set with many more variables than samples (as typical -omics data sets are), on which other methods mentioned in this review (e.g. MANOVA) cannot be used. However, the results on this low-dimensional data are easier to explain and visualize, and the procedure is essentially the same. Examples of application of ASCA to a high-dimensional data set can be found elsewhere [10,38].

### 3.1 PCA and ASCA on raw data

**Fig. 3:**
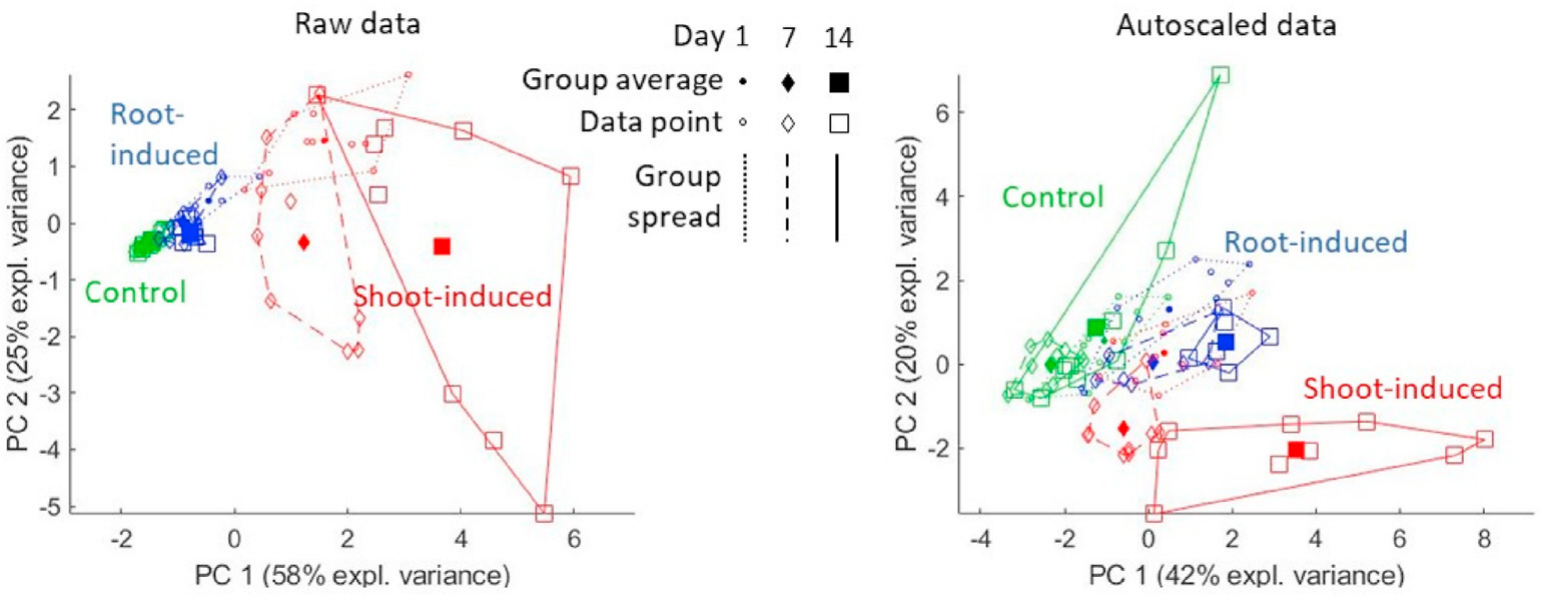
PCA scores plot of the ecotoxicology data as raw (left) and scaled (right).

First, we performed PCA as explorative analysis and the scores of PC1 against PC2 for raw and autoscaled data are plotted Fig. 3. In both cases, an effect of the treatment factor is clearly visible by the separation of the colors. The raw data for the shoot-induced group show a much larger variation than the other two groups, followed by the root-induced and controls. A U-shaped time pattern can arguably be discerned for the non-control samples, especially in autoscaled data. However, these plots do not provide a quantitative assessment of the significance of these time and treatment effects, nor of any treatment-specific dynamics stemming from an interaction. Moreover, the PCs in question may be highly influenced by the variability of the replicates, especially from shoot-induced samples, although this variability is not necessarily related to the factors under investigation.

Therefore, to focus the analysis on the effects of the specific factors of treatment, time and their interaction, we built an ASCA model as in (8), with *a* and *b* indicating treatment and time, respectively, and using type-III sum-of-squares correction for unbalanced data.

Fig. 4a visualizes the ASCA results to the raw data (i.e. without scaling). The effect of treatment can now be observed even more clearly than with normal PCA. A slight effect of time can be observed as well, although there is still a considerable overlap between the different day-groups. No strong group-related differences are visible for the time-treatment interaction. The residuals matrix explains a rather large percentage of the total variance (34%), but a PC score plot of these residuals (see Fig. 4b) reveals no apparent structure, suggesting that this high variance is due to experimental uncertainty, especially of shoot-induced samples after 14 days, rather than underlying data patterns that were not captured by the model. Inspection of the biplots of all these submodels (rightside column of Fig. 4a) reveals a strong alignment of loadings with glucosinolates such as NEO and GBC, and to a lesser degree PRO and GBN. These are the variables with highest standard deviation, as can be seen in the right-hand side plot of Fig. 4b. Such variables can easily dominate the multivariate models derived on the relevant data, but are not necessarily the most important with respect to the considered biological phenomenon.

**Fig. 4a:**
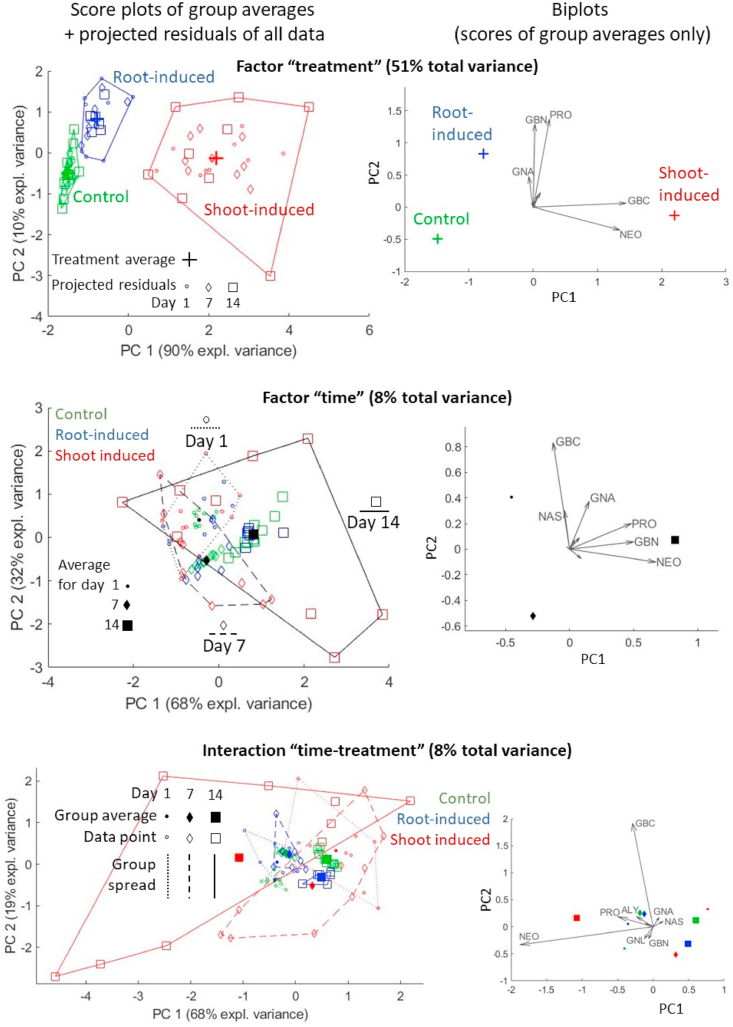
ASCA model for raw (unscaled) data. Left: score plots with projected residuals. Right: biplots; the labels of the variables with smallest loadings have been omitted to avoid overcrowding.

**Fig. 4b:**
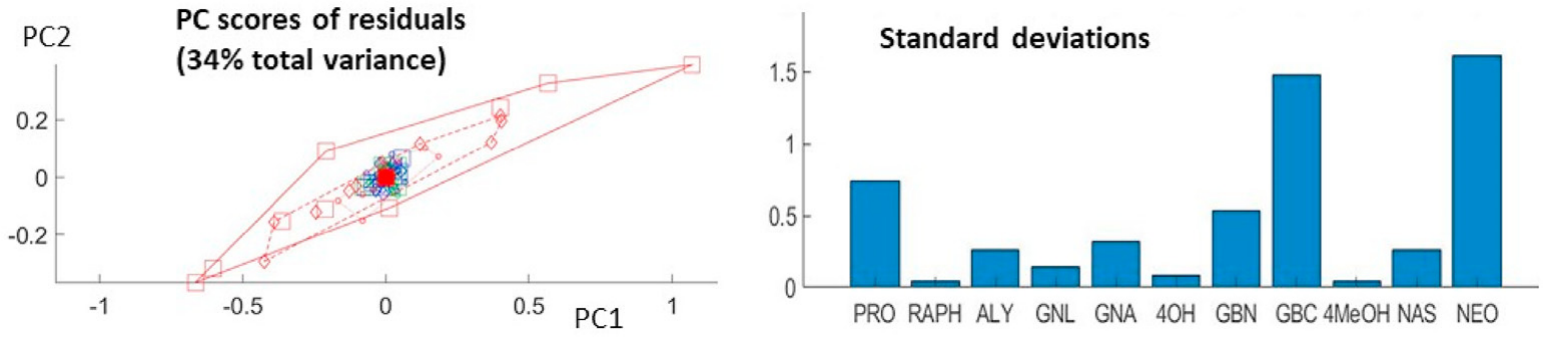
Left: score plot of the PCA on the model residuals. Right: the standard deviation for each variable.

### 3.2 ASCA on scaled data

**Fig. 5:**
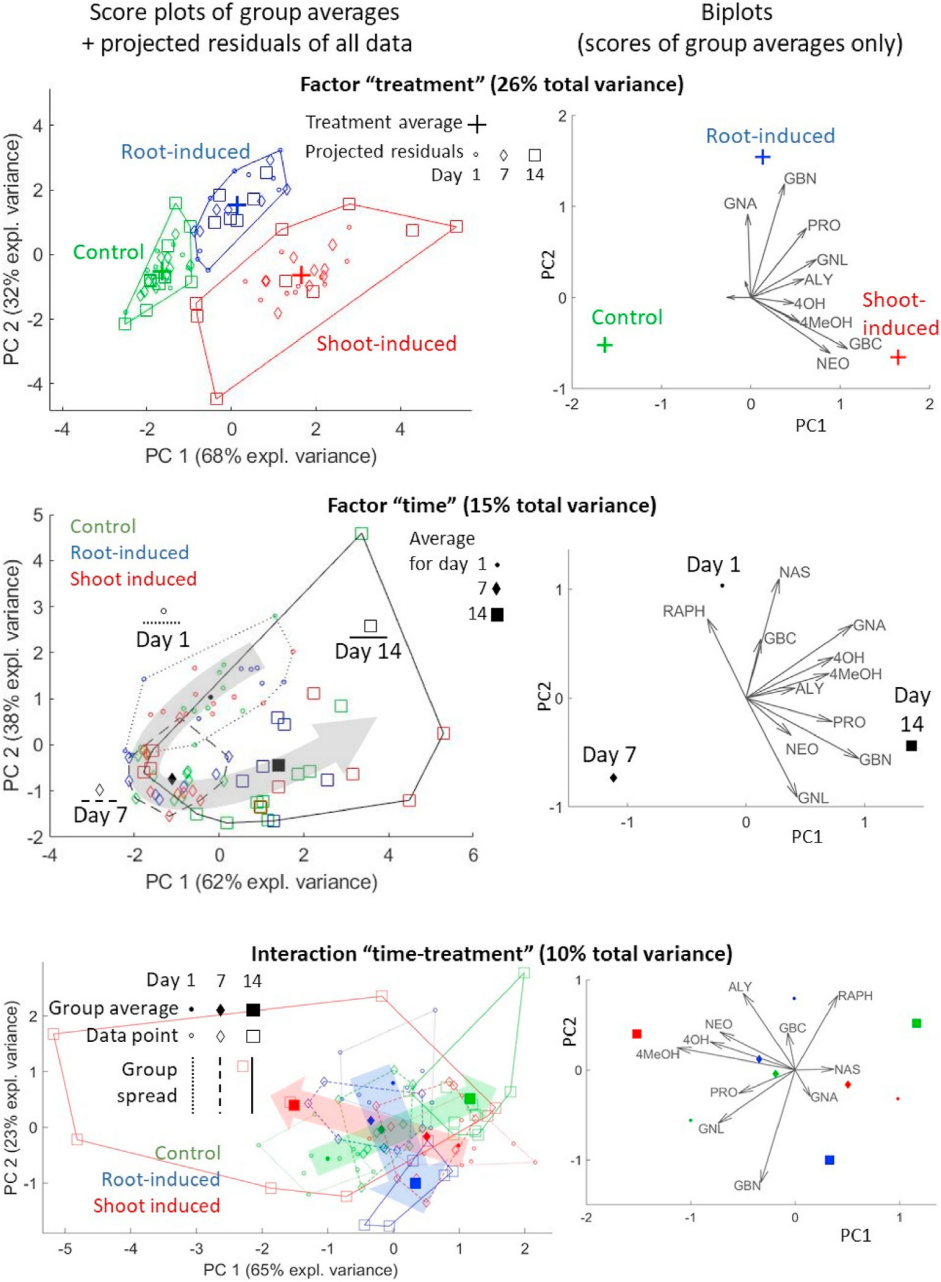
ASCA model for scaled data. Left: score plots with projected residuals. Right: biplots. Dynamic trajectories, both general and treatment-specific, are highlighted by arrows in the score plots.

In order to prevent this oversize influence by responses with high within-group variances, we built a second ASCA model on the autoscaled data, i.e. in which each variable was scaled by its standard deviation. The resulting loadings, plotted on the right-hand side of Fig. 5, are more evenly distributed among all glucosinolates and their sizes are not linked to the within-group variation anymore. The effect of scaling was to remove the absolute concentration as a primary scale of importance, thereby facilitating a view on lower-abundant and perhaps higher-bioactive compounds. For example, compounds such as RAPH and 4MeOH, which were almost invisible in the loadings of Fig. 4a, Fig. 4b, now appear as main contributors in some of the ASCA submodels. The new score plots also allow a better elucidation of the plant dynamics, both in terms of overall time effect (the U-shaped pattern is more clearly visible in the score plot of the time factor) as well as treatment-specific response. Indeed, in the interaction submodel it can be observed that the shoot-induced group shows large dynamic differences along PC1, whereas most of the variability of the root-induced group is expressed by PC2. A permutation test confirms that both main effects and their interaction described by this model are significant, with *p* < 0.001 in all cases. Therefore, it can be concluded that different glucosinolate combinations are produced by the plant depending on where the Jasmonic acid treatment is applied.

A full interpretation of the interactions from a score plot like in Fig. 5 (bottom row) is usually not intuitive, because such submodel represents the deviation of a particular group from the overall effects of the other two factors. In particular, this plot tells that the dynamic response of root-induced samples ends in higher GBN and lower ALY than the overall time effect, while shoot-induced samples develop higher-than-average 4MeOH and 4OH (and other glucosinolates according to their respective loadings); in comparison, the control group has lower GNL and PRO after 14 days. It is important to stress that, since the factor levels are different for every group, the interaction score plot cannot be used to assess similarity of samples based on distance between points, as typically done when reading score plots.

**Fig. 6:**
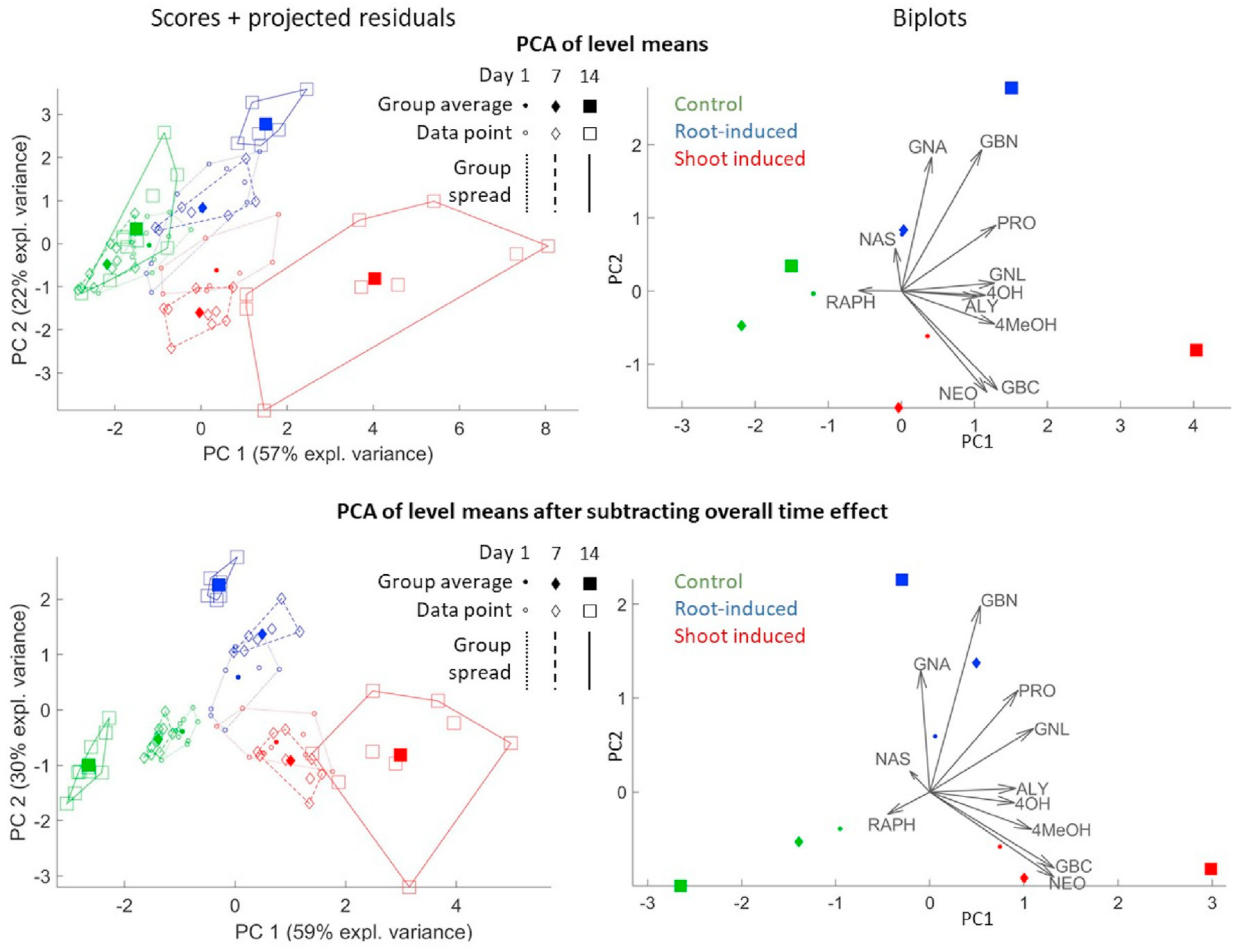
PCA on the level means, scaled data. Left: score plots with projected residuals. Right: biplots. Top half: level means. Bottom half: level means after subtracting the effect matrix for the time factor.

For this reason, it is recommended that the interactions plot be examined together with a more naïve representation of the data set, such as selected raw data or the score plot of a normal PCA. Here we propose to perform a PCA of the group means with projection of the residuals, analogous to the ASCA score plots shown in the paper. The resulting plot, shown in the upper half of Fig. 6, is similar to the normal PCA in Fig. 3, but the directions of the PCs are not influenced by the within-group variation of the data. It is equivalent to an ASCA model built with a single factor containing a different level for each time-treatment combination. Such plot reveals that the samples are, as expected, most similar at the start of the treatment (day 1) and then proceed on diverging paths. Some of the patterns observed in the interaction plot are also visible here, such as the higher values of 4MeOH for shoot-induced samples after 14 days. However, since this plot is so heavily influenced by the main overall trends in the data, it may be difficult to describe the differences in dynamic response in full detail.

For that purpose, it can be helpful to build a PCA model on the level means after subtracting the effect matrix for the time factor, thus removing the common temporal pattern among the groups. This procedure is equivalent to the following ASCA model:

(20) $$X=X_{m}+X_{b}+X_{a+ab}+X_{e},$$

The *(a+ab)* submodel, plotted in the bottom half of Fig. 6, combines the treatment factor with the time-treatment interaction (with *a* and *b* defined as in Section 3.1), and hence focuses on the differences between the groups on each day. This approach can be seen as a compromise between the level of detail of a pure-interaction model and the interpretability of a normal PCA. Such types of models can also be made combining other factors and interactions, depending on the particular aspect that wants to be examined. In the bottom plots of Fig. 6, the points corresponding to the same day (1, 7 or 14) can now be compared with each other as in a normal score plot. They show very clearly that the different treatment groups become more dissimilar with passing days and, compared to the previous plot, it is easier to see along which glucosinolate species this dissimilarity is expressed.

**Fig. 7:**
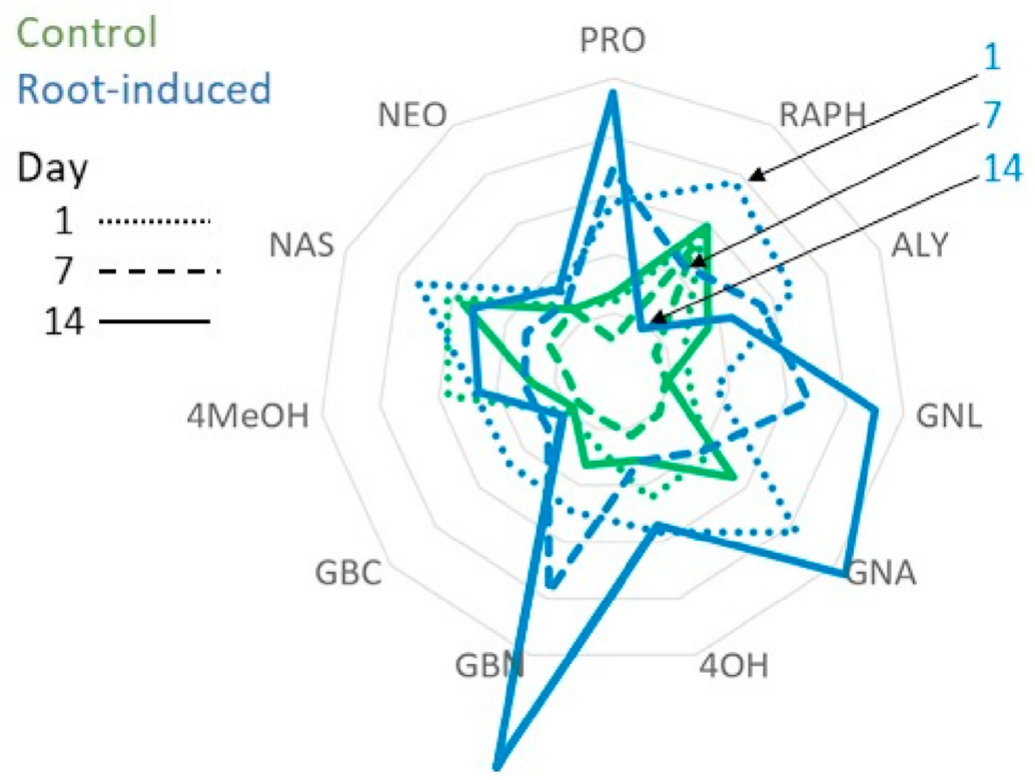
Radar plot of the day-averages of scaled data for the control and shoot-induced groups, respectively. The decrease of RAPH throughout the days is highlighted by the arrows.

It important to point out that all differences observed in these plots are expressed in relative terms. For instance, does RAPH increase over time for the control group or does it decrease for all the others? In light of this, it is good practice to look back at the original data after building the ASCA model. Fig. 7 shows the level averages for each variable as a radar plot; the shoot-induced group is omitted to avoid overcrowding. In this case, it can be clearly seen that the control group remains rather constant, while e.g. the RAPH variable of the shoot-induced group decreases considerably with time.

## 4 Methods related to ASCA

We already pointed out at the beginning of the paper that ASCA is not the only multivariate data analysis approach which takes the experimental design into account. This section provides alternative approaches that are closely related to ASCA, aimed at giving the reader a guideline on when it is appropriate to look for alternatives. For clarity, the methods described below have been subdivided into two broad categories: those that involve a different dimensionality reduction and those that apply a different data decomposition as compared to ASCA. It must be noted that in some cases this distinction is not perfectly applicable, as a method may have aspects of both categories.

### 4.1 Methods with different dimensionality reduction

**Fig. 8:**
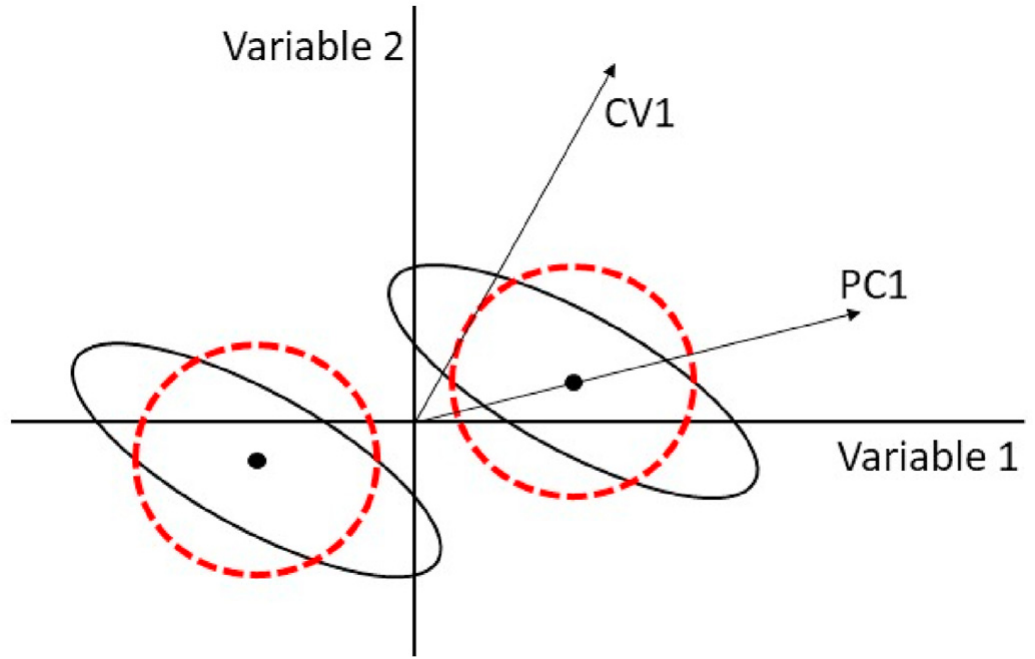
Illustration of the difference between ASCA and MANOVA on a simple example with two variables and two levels. The black dots and the black ellipses indicate the group means and the data spread around these means, respectively. However, ASCA assumes that this spread is distributed along the red dashed circles. Consequently, the PCs can be considerably different than the CVs calculated by MANOVA. (For interpretation of the references to color in this figure legend, the reader is referred to the Web version of this article.)

As mentioned in the Introduction, an earlier way of analysing multivariate data with an underlying experimental design is provided by MANOVA [8]. Like ASCA, it first partitions the data matrix X according to expression (7), then applies a dimension reduction step to each effect matrix while also taking the residual matrix (i.e. within-group variation) into account. However, whereas ASCA uses a PCA to highlight between-group differences assuming uncorrelated (independent) variables with respect to within-group differences, MANOVA takes the within-group covariance matrix into account, i.e. the shape of the data cloud around the level means, as visualized in Fig. 8. Its output includes vectors known as Canonical Variates (CV, analogous to PCA loadings), which indicate the directions along which the between-group differences are maximized relative to the within-group variation [8]. These canonical variates are identical to those obtained by applying Fisher linear discriminant analysis (LDA) to the matrix $X^{*}=X_{f}+X_{e}$ [39], where $X_{f}$ and $X_{e}$ are defined as in (7) and *f* indicates a main or interaction effect. For the statistical validation, several hypothesis tests based on the canonical variates’ eigenvalues (equivalent to PCA scores) have been proposed, such as Wilk’s Lambda, Pillai’s trace, Hoteling’s Trace and Roy’s greatest root [35].

MANOVA can in certain cases highlight the effect of a factor with greater power than ASCA. Indeed, the assumption of variable independence (visualized in Fig. 8 as circular instead of diagonally oriented ellipses) causes the ASCA loadings to be more closely related to univariate test statistics applied to each variable separately [12]. However, as already mentioned MANOVA cannot be used for data with a higher number of variables than observations, because in such case the inverse of the within-group dispersion matrix cannot be computed [40].

The simplest way to solve this problem is to reduce the data dimension using PCA and apply MANOVA to the scores of the first few PCs [41], in what is referred to as PC-MANOVA. A refined version, known as 50/50 MANOVA, also allows for automatic selection of the number of PCs [42]. Compared to ASCA, the order of variance partition and dimension reduction are here reversed, with the advantage that (M)ANOVA is performed on a low-dimensional data set that enables use of all the traditional tools of statistics, e.g. known null distributions or incorporation of random effects. However, the initial dimension reduction ignores the experimental design and risks excluding relevant PCs that do not explain a large amount of data variance.

Another approach to overcome the limitations of both ASCA and MANOVA was proposed by Engel et al. who combined both methods into what is referred to as regularized MANOVA (rMANOVA) [12]. This combination is realized by shrinking the within-group covariance matrix [40], using a weighted average of the within-group covariance matrices of ASCA and MANOVA, determined in a data driven fashion. This weighted estimate of the within-group covariance is then used in (13) and subsequent computations are performed as in MANOVA. Since the null distribution of the Wilk’s lambda statistic is unknown, significance testing is carried out by means of a permutation test. Like ASCA, rMANOVA is applicable to high-dimensional data sets, but it also takes possible correlations among variables into account when considering the within-group variation.Whereas ASCA applies PCA to $X_{f}$ matrices, several methods instead apply it to the residual-augmented matrix $X^{\text{∗}}=X_{f}+X_{e}$ [43,44]. This approach is known as ANOVA-PCA, or APCA (not to be confused with PC-ANOVA). By incorporating the residuals into the effect matrix, only significant effects are likely to produce clear separations in the score plots. However, this approach may miss subtle yet significant effects that are masked by noise or large within-group variation from $X_{e}$. On the other hand, modelling directly the $X_{f}$ matrix as done by ASCA gives greater power in highlighting such effects [21]; this comparison is similar to the difference between normal PCA and the PCA of group-means that we discussed in the plant example. There is also an intermediate option between ASCA and APCA [45] that uses a reduced residuals matrix, obtained by subtracting *n* PCs of $X_{e}$, with *n* optimized by a permutation test.

To facilitate the interpretation of the results, perhaps at the expense of analytical detail, the ANOVA Common Dimensions (AComDim) method was developed [46,47]. Like ANOVA-PCA, it is calculated on the residual-augmented matrix, but it has two important differences: it models several effect matrices simultaneously to find joint components in a common representation space, and it uses a variance-covariance matrix based on samples instead of variables.

Further ways of performing dimension reduction after partitioning the data variance are based on Partial least squares – discriminant analysis (PLS-DA) [[48], [49], [50], [51]]. In particular, PLS-DA is applied to $X^{*}$ (defined as above) to build a classification model in which each class is represented by a level of the factor under consideration. Results can be analysed employing substantially the same tools developed for PLS, such as score plots and variable importance measures (e.g. target projection [48]). Significance testing can be done using the cross-validated classification accuracy as a test statistic, estimating its null distribution by means of a permutation test. This approach takes into account the within-group covariance, but can nevertheless be applied to high-dimensional data. It was also demonstrated by El Ghaziri et al. that ANOVA-PLS can be viewed as a compromise between ASCA and ANOVA-PCA [50].

Further developments included the application of PLS-DA to several effect matrices simultaneously, e.g. $Y^{**}=X_{f_{1}}+X_{f_{2}}+X_{e}$ [49], as well as the use of a kernel-based multiblock Orthogonal Partial Least Squares (AMOPLS) [51]. The latter method obtains a general model based on all effect matrices, rather than one separate PLS-DA model for each effect matrix. Some authors have also proposed using the data matrix to predict the design matrix, thus inverting the roles of D and X [8,41]. This approach does not require any partitioning of the data according to the experimental design before building the PLS model. Its overall performance can be assessed by cross-validation, but it is difficult to deduce the significance of specific main effects or interactions [41].

The dimensionality reduction step does not necessarily have to be based on a bilinear model like the those described above. For instance, the interactions terms in an experimental design can also be described by a multiplicative trilinear model such as PARAFAC, which may provide additional insight into the underlying patterns between two factors. Jansen et al. showed an implementation of PARAFAC into ASCA (named PARAFASCA) applied to a toxicology study that uses metabolomics analyses [52]. Guisset et al. used another metabolomics data set to compare ASCA and APCA with PARAFASCA, AComDim and AMOPLS, concluding that they are all suitable for the considered analysis, but that their different type of outputs may make the interpretation of the results easier in certain cases than in others [53].

### 4.2 Methods with different data decompositions

As mentioned in section 2.1, sum-to-zero constraints are normally applied in ASCA to ensure uniqueness in the parameters of the final model, thus describing every effect as deviation from the overall mean μ. However, in certain situations it may be more insightful to use other constraints or linear decompositions that better reflect a specific variability of interest. Several of these decompositions are described and compared by Smilde et al. in a generic framework of techniques combining ANOVA with dimensional reduction [11]. They include Principal Response Curves (PRC), a method to study dynamic dose-responses, describing them as deviations of treatment groups from the control group, measured at the same time-points [40]. Another method named Scaled-to-Maximum, Aligned, and Reduced Trajectories (SMART) [54] is used in dose-response studies with repeated measures and expresses all samples as deviations from the pre-dose samples of the corresponding individual, thereby removing constitutive biological variability between biological replicates.

A similar objective is pursued by REP-ASCA, which models the repeatability error from a separate set of repeated measures and performs an orthogonal projection in the row-space to reduce the repeatability error of the original dataset; ASCA is then performed on the resulting orthogonalized dataset [55]. In a NIR study of coffee beans, this method was able to increase the power of ASCA and reveal spectral features in the loadings of factors of interest that were previously covered by experimental noise.

On the other hand, it can also be insightful to focus precisely on the analysis of the residuals matrix $X_{e}$, since patterns or sub-groupings observed in the residuals may reveal an underlying data structure that was not accounted for in the experimental design. The most straightforward way to realize this is a PCA on $X_{e}$, as we did in the plant example. However, there are also methods specifically devised for this purpose such as SCA-IND, which models the variability across individual samples for each group by combining SCA with Individual Differences Scaling. Jansen et al. applied it to the same plant data of the previous section, identifying early and late responders in the root-induced group, as well as two major response chemotypes for the shoot-induced group [56].

When analysing data sets from different compartments or analytical platforms, which nevertheless share the same underlying experimental design, it is of particular interest to highlight the variation common to all sets as opposed to the variation specific to a particular one. Penalized Exponential ANOVA simultaneous component analysis (PE-ASCA) realizes this task by first decomposing the data matrix into common and distinct variation, and subsequently applying ASCA to each resulting submatrix [57]. A similar objective is also tackled by Huopaniemi et al. [58] using a Bayesian approach.

## 5 Conclusion

The ever pressing need to account for the experimental design when modelling multivariate data has spurned the development of a variety of approaches to deal with this task, which have been surveyed in this tutorial review. Despite this prolific research activity, ASCA still stands out as one of the methods applicable in the widest range of cases, and whose utilization and interpretation is relatively straightforward. This paper explains step-by-step its main principles and use, by means of a couple of simple examples that nevertheless demonstrate the clear advantage of incorporating information on the experimental design into a chemometric model. These advantages are even more evident when analyzing complex designs and/or high-dimensional data, for which not all ANOVA-related methods are applicable. Special importance was placed into showing how to interpret results correctly, providing various graphical tools that assist this task in the most intuitive way possible. They are not meant as the only possible approach, but as a framework from which the reader is encouraged to develop further perspectives adapted to the problem under study.

Moreover, this work provides an overview of alternative methods to ASCA, describing the situations in which they might be more suitable, e.g. to take the within-group variance into account, to employ an ad-hoc data decomposition or to deal with mixed designs. This information constitutes a simple yet comprehensive guide that can help chemometricians and data analysts select the best approach to build models that include knowledge of the experimental design.

## 6 Software

Several online packages for ASCA are available in R and Matlab, the languages used to perform the calculations discussed in this paper:

- https://cran.r-project.org/src/contrib/Archive/MetStaT/
- http://www.bdagroup.nl/content/Downloads/software/software.php

There are also stand-alone platforms that do not require any programming:

- PLS_toolbox: http://www.eigenvector.com/software/pls_toolbox.htm
- MetaboAnalyst [59]: https://www.metaboanalyst.ca/

ASCA can also be performed using the rMANOVA code (https://github.com/JasperE/regularized-MANOVA), which also contains sums-of-squares corrections for analysis of unbalanced data.

It is worth noting that none of the software above can perform all of the operations shown in this paper. For certain advanced or ad-hoc applications, a little programming is necessary.

## Declaration of competing interest

The authors declare that they have no known competing financial interests or personal relationships that could have appeared to influence the work reported in this paper.

## Figures and Tables

**Table A1:** Usual constraints and parameter estimators for the two-way ANOVA model for balanced designs.

Parameter	Constraint	Estimator
μ	−	$\hat{\mu }={\bar{y}}_{\ldots }$
$\alpha_{i}$	$\overset{I}{\underset{i=1}{\sum }}\alpha_{i}=0$	${\hat{\alpha }}_{i}={\bar{y}}_{i..}-y_{\ldots }$
$\beta_{j}$	$\overset{J}{\underset{j=1}{\sum }}\beta_{j}=0$	${\hat{\beta }}_{j}={\bar{y}}_{.j.}-{\bar{y}}_{...}$
${\left(\alpha \beta \right)}_{ij}$	$\overset{I}{\underset{i=1}{\sum }}\overset{J}{\underset{j=1}{\sum }}{\left(\alpha \beta \right)}_{ij}=0$	${\hat{\alpha \beta }}_{ij}={\bar{y}}_{ij.}-{\bar{y}}_{i..\;}-{\bar{y}}_{.j.}+{\bar{y}}_{...}$
$\varepsilon_{ijk}$	−	${\hat{\varepsilon }}_{ijk}=y_{ijk}-{\bar{y}}_{ij}.$

## Appendix

The value ${\bar{y}}_{\ldots }=1/\left(IJK\right)\underset{ijk}{\sum }y_{ijk}\;$ is sample mean of the response (average over all observations), ${\bar{y}}_{i..}=1/\left(JK\right)\underset{jk}{\sum }y_{ijk}$ is the sample average of the response of all observations in temperature-group i, ${\bar{y}}_{.j.}=1/\left(IK\right)\underset{ik}{\sum }y_{ijk}\;$ is the sample average of all observations in catalyst group j, and ${\bar{y}}_{ij.}=1/\left(K\right)\underset{k}{\sum }y_{ijk}$ is the sample average of the observations in temperature/catalyst-group ij.
